# Special Convening and Listening Session on Health Equity and Community Outreach and Engagement at National Cancer Institute-Designated Comprehensive Cancer Centers

**DOI:** 10.1089/heq.2020.0155

**Published:** 2021-02-26

**Authors:** Patricia M. Doykos, Moon S. Chen, Karriem Watson, Vida Henderson, Monica L. Baskin, Sarah Downer, Lauren A. Smith, Neeraja Bhavaraju, Samantha Dina, Christopher S. Lathan

**Affiliations:** ^1^ Bristol Myers Squibb Foundation, New York, New York, USA.; ^2^ UC Davis Comprehensive Cancer Center, Sacramento, California, USA.; ^3^ University of Illinois Cancer Center, Chicago, Illinois, USA.; ^4^ O'Neal Comprehensive Cancer Center at the University of Alabama at Birmingham, Birmingham, Alabama, USA.; ^5^ The Center for Health Law and Policy Innovation, Harvard Law School, Cambridge, Massachusetts, USA.; ^6^ FSG, Boston, Massachusetts, USA.; ^7^ Dana-Farber Harvard Cancer Center, Boston, Massachusetts, USA.

**Keywords:** health equity, community outreach and engagement, cancer center

## Abstract

In recent years, the cancer research and care community has been more attuned to health equity, increasingly pursuing coordinated and comprehensive action to achieve equitable health outcomes. In addition to its support of a joint research agenda for health disparities in 2017, the National Cancer Institute (NCI) has demonstrated its commitment to addressing health inequities with its 2012 requirement for cancer centers to define and address the needs of a local “catchment area” and the 2016 mandate for Community Outreach and Engagement (COE). With several years of experience with the COE requirements, there is an opportunity to reflect on the experience to-date and identify opportunities to bolster the impact of COE on equitable cancer outcomes for the future. To do so, the Bristol Myers Squibb Foundation (BMSF) hosted a special convening and listening session in April 2019. The session agenda was cocreated by BMSF and NCI leaders and staff. It brought together 41 individuals, including representatives from the NCI Cancer Centers Program, Division of Cancer Control and Population Health and Center to Reduce Cancer Health Disparities, 22 NCI-designated, emerging or affiliated comprehensive cancer centers, and the broader cancer community. This article captures key themes from that meeting, including an overview of current COE efforts, with a deeper look at how four cancer centers are embedding health equity and COE efforts into their institutions and work, and the successes and challenges they have encountered.

## A Convening on Health Equity Efforts at Cancer Centers and the Evolving National Cancer Institute's Community Outreach and Engagement Requirements for Designated Comprehensive Cancer Centers

In recent years, the cancer research and care community has been more attuned to health equity, increasingly pursuing coordinated and comprehensive action to achieve equitable health outcomes. In addition to its support of a joint research agenda for health disparities in 2017, the National Cancer Institute (NCI) has demonstrated its commitment to addressing health inequities with its 2012 requirement for cancer centers to define and address the needs of a local “catchment area” (CA) and the 2016 mandate for Community Outreach and Engagement (COE).^1,2^

With several years of experience with the COE requirements, there is an opportunity to reflect on the experience to-date and identify opportunities to bolster the impact of COE on equitable cancer outcomes for the future. To do so, the Bristol Myers Squibb Foundation (BMSF) hosted a special convening and listening session in April 2019. The session agenda was cocreated by BMSF and NCI leaders and staff. It brought together 41 individuals, including representatives from the NCI Cancer Centers Program, Division of Cancer Control and Population Health and Center to Reduce Cancer Health Disparities, 22 NCI-designated, emerging, or affiliated comprehensive cancer centers (CCC), and the broader cancer community.

This article captures key themes from that meeting, including an overview of current COE efforts, with a deeper look at how four cancer centers are embedding health equity and COE efforts into their institutions and work, and the successes and challenges they have encountered.

## Current Community Outreach and Engagement Activity at Cancer Centers

**FIG. 1.**
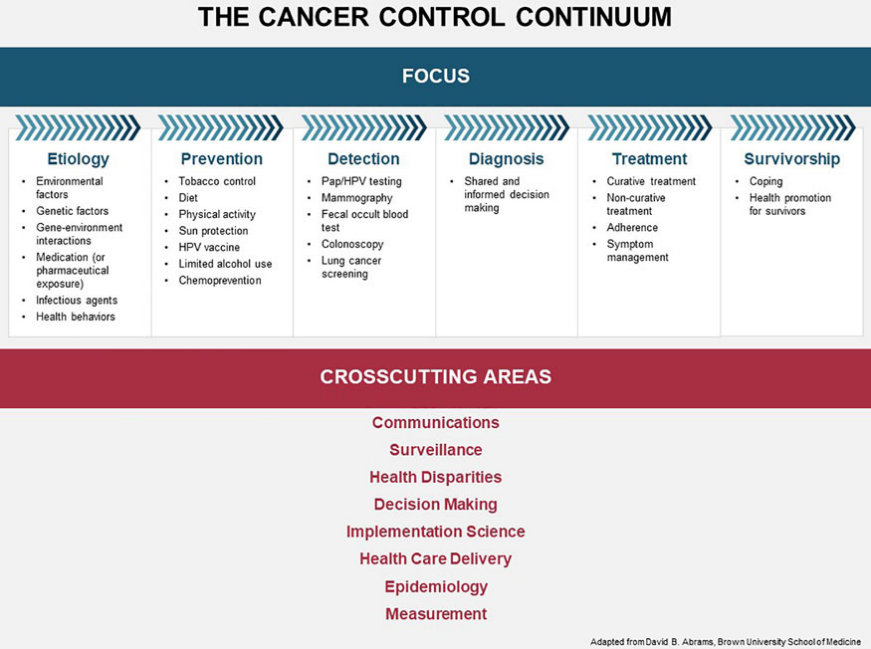
The National Cancer Institute cancer control continuum.

Inequitable health outcomes are the result of historical and structural issues that have created and perpetuated disparities across every stage and aspect of cancer care, from risk factors to diagnosis, treatment, and survivorship. The NCI Cancer Control Continuum (Fig. 1) provides a comprehensive framework to consider the various goals and activities for COE.^3^ Convening participants shared examples of their programs to address disparities across the Cancer Control Continuum, which are summarized below.

### Etiology and research

Cancer centers are increasingly aligning research priorities to community needs. This includes better identifying and understanding disparities by analyzing data disaggregated by race and ethnicity as well as other demographics, conducting implementation science research to inform improvements in care and treatment for medically underserved populations, and ensuring diversity in clinical trial participation.^1^

The *Sidney Kimmel Cancer Center—Jefferson Health* in Philadelphia, for example, uses patient residence zip code to anticipate risk factors for cancer that should inform care and patient education and engagement. *Maine Medical Center* conducts qualitative research with community focus groups to identify factors it should incorporate into care delivery, such as perspectives on fatalism associated with lung cancer and community distrust of the health system.

Many cancer centers are also establishing formal mechanisms to regularly gather community input, and importantly, to share back research findings with the community. For example, *North Carolina's Levine Cancer Institute* has its cancer program development specialists communicate directly with community members to inform its services. The *University of Hawaii* gathers community input on research priorities through processes that align with local cultures and values, and acknowledge the impact of historical trauma through engagement with four community advisory groups, including a Patient Advocacy Council and Native Hawaiian Scientific Advisory Committee.

The *University of Illinois Cancer Center* (UICC) has taken a different approach, creating a talent pipeline for clinical research staff that reflects the diversity representative of its local community and the diversity of skills to deliver on its tagline—“bench to community.”

### Prevention

Recognizing that inequities in health outcomes are often the result of historic structural inequities that manifest in risk factors, cancer centers are increasingly focusing on primary and secondary prevention and early detection in addition to treatment.^4^ Research has shown, for example, that sales of menthol cigarettes, which carry greater lung cancer risk than tobacco cigarettes, were systematically marketed to communities of color.^5^ Addressing this source of inequity requires nonclinical action, including community outreach and mobilization to advance policies that address structural factors, education, and the community conditions that enable risk reduction and behavioral change.

The National Outreach Network (NON) supports a strengthening of community outreach capacity by working through community health educators located at NCI Cancer Centers.^6^ It is expected that NON community health educators, will enhance the development and dissemination of culturally appropriate, evidence-based cancer information to members of underserved communities, including rural and racially and ethnically diverse populations. Over 16 of the cancer center presenters received support from NON.

An example of community outreach is *Yale Cancer Center's* robust lung cancer prevention program that includes tobacco cessation, weight management, and free lung cancer screening events in community venues, such as faith-based organizations. *O'Neal Comprehensive Cancer Center at the University of Alabama at Birmingham* has created a regular “No Menthol Sunday” event to raise community awareness of the dangers of menthol cigarettes and other tobacco products (e.g., e-cigarettes).

Cancer centers are also increasingly lending their expertise to further the efforts of existing coalitions and engaging in policy to reduce disparities in prevention. For example, *UC Davis Comprehensive Cancer Center* has advised on regulations on flavored or menthol tobacco to prevent exposure to these risk factors in their community.

### Detection and diagnosis

To mitigate disparities in the quality and timeliness of cancer screening and diagnosis, cancer centers are expanding screening programs and the supportive services needed to make them effective.^7^

*MD Anderson Cancer Center*, for example, collaborates with Project ECHO to build capacity of primary care providers in rural Texas and along the Texas-Mexico border to increase human papillomavirus (HPV) vaccination uptake and provide cervical cancer screening, which can improve access particularly for migrant Latina women who find travel cost-prohibitive or too high-risk. Similarly, the *Penn Medicine's Abramson Cancer Center* works with community partners to improve the reach of its breast cancer screening program, incorporating tele-translation services to ensure effective communication with linguistically and ethnically diverse patient populations around diagnosis and treatment and care decision-making.

The *University of Kentucky Markey Cancer Center* uses a Proactive Office Encounter approach to identify increased risk factors among patients before a patient visit and implement a tailored, evidence-based approach to cancer screening and early detection. The *Chao Family Comprehensive Cancer Center at UC Irvine* leverages culturally competent community navigators to increase screening of Asian American and Latino MediCal beneficiaries. The UICC uses evidence-based patient navigation to mitigate barriers to screening access and awareness, prevent delays in cancer care, and provide cancer education for breast, colorectal, cervical, and lung cancer. In an effort to create large-scale change, the *UCSF Helen Diller Family Comprehensive Cancer Center* serves as the backbone organization facilitating multiple task forces to enable collaboration with community organizations and government agencies to improve prevention and early detection across five cancer types in San Francisco.

### Treatment

To reduce disparities in treatment outcomes, cancer centers are pursuing partnerships with community-based health care organizations to streamline care pathways and provide more holistic care.^1^ *Dana Farber Cancer Center*, for example, has established partnerships with local Federally Qualified Health Centers (FQHCs) and primary care providers to support timely cancer screening and faster referrals for follow-up and resolution. The *Ralph Lauren Center for Cancer Care*, *now a formal entity of Memorial Sloan Kettering Cancer Center* in New York City, focuses its efforts on “in reach” to educate its established network of primary care partners on screening guidelines, and on outreach to provide social support to cancer patients (housing, food, financing) that enable them to complete courses of care.

### Survivorship

Survivorship factors can be substantial drivers of cancer inequities. For example, they can result in impacts on a survivor's ability to maintain access to health insurance if unable to work or travel to follow-up appointments.^8^ Cancer centers are increasingly cognizant of the needs of cancer survivors, including effective clinical transition as well as support for ongoing self-care, behavior change, education in whole person health, and maintenance. For example, the FOSTER program at the *University of New Mexico Comprehensive Cancer Center* supports effective transitions for survivors moving from cancer center oncology care to primary and community-based care setting in rural areas in New Mexico.

### Cross-cutting areas

COE interventions can also cut across the Cancer Control Continuum. *Project ECHO*, for example, leverages its tele-mentoring and collaborative care model to create connections between cancer center-based multidisciplinary specialist teams and community oncology and primary care physicians to build local cancer care capacity, especially in rural areas, for prevention, diagnosis, treatment, and survivorship. Likewise, the *Cancer Institute of New Jersey* is building a platform to disseminate evidence-based practices and tools to health care providers and community organizations throughout the state aimed at increasing prevention, screening, and early detection efforts across the most prevalent cancers.

## Health Equity and COE at Four Comprehensive Cancer Centers

COE as mandated by the NCI is just one of many ways, in which cancer centers are increasingly working toward equitable cancer outcomes. To provide perspective on how NCI-supported COE activities integrate with broader health equity efforts at cancer centers, three NCI-designated CCCs and one emerging center presented overviews of their institutions' commitment, governance, programs, and challenges related to COE. For these centers, COE is a not a vertical program area, but rather an essential cross-cutting and foundational support undergirding high-quality research and clinical care.

➢  The *Dana-Farber Harvard Cancer Center* (DFHCC) serves the Greater Boston Area and embeds a health equity approach into research and care delivery. The Center's Office of Cancer Equity and Engagement functions as a coordinating structure for a variety of DFHCC health equity and COE programs. Despite this structure at DFHCC and significant local and regional multisector cancer control efforts, low-income, immigrant, and minority populations in DFHCC's CA continue to experience disproportionate cancer burden.

In a major effort to address these inequities, DFHCC conducted implementation science research to understand cancer disparities in the local population and identify barriers to access to care. DFHCC then engaged local health system leaders and community members to codesign interventions aimed at accelerating cancer diagnosis and linkage to care. The resulting intervention consists of DFHCC staff embedded within a local FQHC and a community hospital to provide education, initial cancer screenings, streamlined referral systems between primary care providers and oncology specialists, second opinion and treatment plan confirmation services, and multilingual nurse navigation to assist patients through screening and treatment protocols. These efforts have led to increased clinical trial enrollment of minority populations and clinical quality outcomes, including decreased time to resolution of cancer diagnoses.

While DFHCC has implemented effective COE programs, continued institutional support, financial resources, and systems to enable collaboration between DFHCC, FQHC partners, and local primary care providers will be needed to sustain and scale the initiative and realize its full potential impact on patient and CA outcomes.

➢ The *O'Neal Comprehensive Cancer Center* at the University of Alabama at Birmingham has a long legacy of leadership in cancer health equity and innovating evidence-based models of care and support for rural and low-income communities. O'Neal led the NCI-funded Deep South Network for Cancer Control and has an 11-person COE team with members based both at the cancer center and in local communities. Its CA is the state of Alabama—a state with one of the highest cancer mortality rates in the country and where a history of structural violence and systematic marginalization of populations of color has contributed to significant racial disparities in health outcomes and fostered distrust of health systems in communities of color.

O'Neal works to address these challenges in its approaches to lung, breast, and prostate cancer for rural communities and communities of color by leveraging a network of community health advisors to reach those at high-risk of cancer with disease information, prevention support, cancer screening, and treatment. It also engages a Community Advisory Board to provide input on aligning research plans with community needs and interests.

The Center's research includes behavioral and environmental risk factors, with current studies focused on tobacco cessation, healthy eating, and food insecurity. O'Neal also implements health policy recommendations throughout its CA such as the NCI's *Screen to Save* initiative as well as evidence-based practices from the American Cancer Society's Cancer Action Network.^9^ Its shared facility for clinical trial recruitment and COE has led to increased participation in clinical trials from communities of color.

Despite strong institutional support and progress in health equity, O'Neal remains challenged by the size and diversity of its CA, lack of insurance among individuals in need of treatment, and maintaining COE programs beyond dedicated grant funding.

➢ The *UC Davis Comprehensive Cancer Center* (UCD) serves 19 inland northern California counties, the size of West Virginia, with a population of 5 million people. The area's population is majority-minority, with greater proportions of Hispanics, Asian Pacific Islanders, and Native Americans than non-Hispanic whites. Within its CA, there is a disproportionately high rate of liver cancer mortality among Hispanic and Pacific Islander communities.

UCD's multipronged approach focuses on prevention, control, and improved quality of life, while incorporating COE and research elements. UCD is engaging with the local community to increase colorectal cancer screening by implementing NCI's *Screen to Save* program in partnership with local FQHCs and leveraging input from a community advisory board to inform its research agenda. UCD is also studying HPV vaccine uptake factors and is combining population science and therapeutics in its research by securing and testing tumor tissue samples from underrepresented racial and ethnic populations in gastric, liver, and lung cancer to study differences in response to therapies. UCD's research has also translated into policy recommendations, and UCD recently held a public forum on use of flavored tobacco, which informed policies restricting access to this carcinogen.

The success and breadth of UCD's COE efforts within its diverse CA are, in part, a result of the recent NCI COE mandate, which provided support for the associate director of COE position. Importantly, this position reports directly to the cancer center director. Despite this progress, UCD continues to find it challenging to prioritize among COE efforts to best meet the diverse needs within its CA.

➢ The UICC is pursuing NCI CCC designation while defining its mission as being a community-focused cancer center. It aims to inclusively serve its community from “bench to bedside” by conducting research on health inequities, integrating services directly with the 13 FQHCs that it owns as well as safety net hospitals, building relationships with community organizations, and improving the diversity of cancer researchers. UICC defines its CA as five counties, including the city of Chicago with disproportionate incidence, morbidity and mortality for cervical, colon, lung, and prostate cancer, particularly among African American populations.

UICC is conducting research on the impact of structural violence on physical health and is leveraging social engagement theory to recruit local African American men as “citizen scientists” to test the accuracy of cancer biomarkers. UICC is also leveraging NCI funding to provide training for faculty and students of a local minority-serving university and partners with local faith, school, and community-based organizations to increase cervical and prostate cancer screening, access, awareness, and navigation services among African American populations.

While UICC has deep leadership commitment to community engagement and addressing health inequities, systemic barriers continue to prove challenging and impede progress; in particular, gaps in insurance coverage for lower income patients and poor coordination within the cancer care system to establish clear care pathways from screening to treatment.

## A Path Forward

Recent experiences have yielded lessons for how cancer centers can effectively pursue health equity in their communities and for how the NCI can be an effective partner in this work. Participants highlighted the COE mandate as an effective way to raise the profile of COE and health equity within their institutions and that resourcing for leadership positions dedicated to COE was particularly helpful in creating change.

Participants also noted that without additional dedicated resources and structural change, sustaining and scaling COE interventions remain difficult. They highlight how the impact of COE activities on disparities can be improved by taking a more explicit health equity approach, addressing structural barriers, providing adequate resources, improving the quality of care by building patient pathways, advancing relevant policies, and ensuring effective evaluation of COE activities and relationships.

This focused dialogue between the NCI, cancer centers, professional and patient advocacy organizations, philanthropy, and communities enabled organizations to share insights and effective practices, identify common challenges and provide input to inform the NCI's COE guidelines, cancer disparities research priorities, funding and engagement mechanisms, and metrics to improve their impact on equitable cancer outcomes. Continuing this dialogue as the field moves forward on health equity will be critical. Even more, it can catalyze the establishment of inclusive research and care as a standard of excellence and enhance the quality of the science and the relevance to the cancer burden of the nation's leading cancer centers.

## Abbreviations Used

BMSF: Bristol Myers Squibb Foundation
CA: catchment area
CCCs: Comprehensive Cancer Centers
COE: community outreach and engagement
DFHCC: Dana-Farber Harvard Cancer Center
FQHC: Federally Qualified Health Center
HPV: human papillomavirus
NCI: National Cancer Institute
NON: National Outreach Network
UCD: UC Davis Comprehensive Cancer Center
UICC: University of Illinois Cancer Center
